# Tumor Lysis Syndrome: Introduction of a Cutaneous Variant and a New Classification System

**DOI:** 10.7759/cureus.13816

**Published:** 2021-03-11

**Authors:** Philip R Cohen, Victor G Prieto, Razelle Kurzrock

**Affiliations:** 1 Dermatology, San Diego Family Dermatology, National City, USA; 2 Pathology, MD Anderson Cancer Center, Houston, USA; 3 Center for Personalized Cancer Therapy, University of California San Diego Moores Cancer Center, La Jolla, USA

**Keywords:** cancer, carcinoma, cutaneous, hematologic, lysis, malignancy, metastatic, solid, syndrome, tumor

## Abstract

Tumor lysis syndrome, an oncological emergency, is characterized by laboratory parameters such as hyperuricemia, hyperkalemia, hyperphosphatemia, and hypocalcemia, as well as renal injury with an elevated creatinine. Tumor lysis syndrome is seen in patients with aggressive malignancies and high tumor burden. More frequently, it occurs in individuals with hematologic malignancies such as high-grade lymphomas (such as Burkitt lymphoma) and leukemia (such as acute lymphocytic leukemia). It also, albeit less commonly, can be seen in patients with widespread solid tumors that are rapidly proliferating and are markedly sensitivity to antineoplastic therapy. Tumor lysis syndrome is usually preceded by cancer-directed therapy; however, the syndrome can present spontaneously prior to the individual receiving malignancy-directed treatment. We reported a man with metastatic salivary duct carcinoma who had cutaneous metastases that presented as carcinoma hemorrhagiectoides. Microscopic examination demonstrated that the metastatic tumor cells had infiltrated and replaced the entire dermis. After the patient received his first dose of antineoplastic therapy, he had an excellent response and the cutaneous metastases developed into ulcers; we hypothesize that most of the dermis, which had been replaced by tumor cells, disappeared as a result of the therapeutic response, and the overlying epidermis became necrotic and shed, leaving an ulcer. His dramatic response to treatment prompted us to propose a new classification of tumor lysis syndrome, which should include the systemic form of the condition as well as the new variant: cutaneous tumor lysis syndrome. We anticipate that, with improvement in targeted therapies, there may be an increase in therapy-associated cutaneous tumor lysis syndrome.

## Introduction and background

Tumor lysis syndrome is an oncological emergency. It most commonly occurs in patients with hematologic malignancies. However, it can be observed in patients with solid tumors, albeit less frequently [1]. Tumor lysis syndrome is currently defined by laboratory and clinical criteria. Most of the patients who experience tumor lysis syndrome have aggressive disease and develop the condition after receiving cancer-directed therapy. Yet, the condition also occurs spontaneously in individuals with untreated cancer [2].

Based on our experience, we suggest a change to the current nomenclature of tumor lysis syndrome classification. We previously observed that the cancer-associated skin lesions in a man with aggressive cutaneous metastatic salivary duct carcinoma developed into ulcers, associated with rapid tumor regression, after one cycle of antineoplastic therapy. We suggest that this clinical phenomenon be referred to as cutaneous tumor lysis syndrome [3-5].

## Review

Tumor lysis syndrome

Tumor lysis syndrome was originally described in hematologic malignancies in 1963 [6]. It more commonly occurs in patients with high-grade lymphomas (such as Burkett lymphoma) and leukemia (such as acute lymphocytic leukemia) [7]. Subsequently, in 1977, the first patient with solid tumor-associated tumor lysis syndrome was described [8]. In both settings, tumor lysis syndrome is typically associated with a rapidly proliferating malignancy in which there is widespread disease and marked sensitivity to antineoplastic therapy [1,2,9,10].

Based on the type of malignant involvement, two subtypes of tumor lysis syndrome are proposed here: systemic tumor lysis syndrome and cutaneous tumor lysis syndrome (Table 1) [1-50]. Systemic tumor lysis syndrome presents with laboratory abnormalities in patients with a high tumor burden. Of relevance, diffuse cutaneous metastases, while rare, are a sign of extensive disease and poor prognosis. When cutaneous metastases show diffuse ulceration, especially in response to therapy and in the presence of tumor regression, we suggest that the condition be designated cutaneous tumor lysis syndrome.

**Table 1:** Classification of tumor lysis syndrome. ^a^Some references present cases with manifestations suggestive of various forms of tumor lysis syndrome ^b^By definition, tumor lysis syndrome should be accompanied by tumor cell death ^c^Spontaneous tumor lysis syndrome is generally idiopathic ^d^Therapy-associated tumor lysis syndrome includes syndrome-related causes such as corticosteroids, embolization, induction chemotherapy, immunotherapy, interferon, monoclonal antibody therapy, radiotherapy, surgery, and tamoxifen

Subtype	References^a^
Systemic tumor lysis syndrome^b^	[1,2,6-26]
Spontaneous (idiopathic)^c^	[1,2,6-8,10-23]
Hematologic malignancies	[1,6,10,12-15]
Solid tumors	[2,7,8,10,11,15-23]
Therapy-associated^d^	[1,2,9-11,15,17,20,21,23-26]
Hematologic malignancies	[1,2,9,10]
Solid tumors	[10,11,15,17,20,21,23-26]
Cutaneous tumor lysis syndrome^b^	[3-5,7,27-50]
Spontaneous (idiopathic)^c^	[7,27-39]
Hematologic malignancies	[27-34]
Solid tumors	[7,35-39]
Therapy-associated^d^	[3-5,27,40-50]
Hematologic malignancies	[27,40-43]
Solid tumors	[3-5,44-50]

Systemic tumor lysis syndrome

Systemic tumor lysis syndrome has previously been referred to as tumor lysis syndrome. However, we suggest that the “systemic” designation be used to differentiate it from cutaneous tumor lysis syndrome. Systemic tumor lysis syndrome is characterized by massive lysis of malignant cells followed by the release of cellular contents, including potassium, phosphate, and nucleic acids into the blood stream. Systemic tumor lysis syndrome is an oncological emergency [1].

Laboratory features of systemic tumor lysis syndrome include at least two of these abnormalities: hyperuricemia, hyperkalemia, hyperphosphatemia, and hypocalcemia. Clinical features of systemic tumor lysis syndrome include at least one of the following: elevated creatinine (1.5 times the upper limit of normal), cardiac arrhythmia, and/or seizures. Systemic tumor lysis syndrome can be preceded by malignancy-related treatment or occur as a spontaneous event. It is generally idiopathic, and the underlying trigger is not known [1,2,6-26].

Spontaneous (Idiopathic) Systemic Tumor Lysis Syndrome

Spontaneous (idiopathic) systemic tumor lysis syndrome is less common than therapy-associated tumor lysis syndrome. However, multiple reports now document its occurrence [1,2,6-8,10-23].

Hematologic malignancies: Spontaneous (idiopathic) systemic tumor lysis syndrome has been observed in patients with leukemia and lymphoma [12-14]. For some of these patients, malignancy may have been recently diagnosed and treatment was being planned [13]. However, in other individuals, idiopathic systemic tumor lysis syndrome was the presenting manifestation of their previously undiagnosed leukemia or lymphoma [12,14].

The patients typically present with constitutional symptoms such as weakness and fatigue, dyspnea, loss of or reduced appetite, and nausea or vomiting; fever and night sweats may be present or absent. Eventually, they seek medical attention, and the abnormal serum chemistry results from preliminary laboratory studies prompt consideration of systemic tumor lysis syndrome. The complete blood cell count or computerized axial tomography examination subsequently disclose aberrations that lead to biopsy of the bone marrow or lymph node or both to establish the diagnosis of the associated hematologic malignancy [1,6,10,12-15].

Solid tumors: Spontaneous (idiopathic) systemic tumor lysis syndrome has also been observed in patients with solid tumors. Similar to individuals with associated hematologic malignancies, spontaneous (idiopathic) systemic tumor lysis syndrome may occur in patients with recently diagnosed, but untreated, visceral cancers, or it may be an initial clinical feature that heralds the diagnosis of an unsuspected solid tumor. Although solid tumor-associated idiopathic systemic tumor lysis syndrome is uncommon, reports of individuals in whom this occurs seem to be increasing [2,7,8,10,11,15-23].

The first patient with solid tumor-associated idiopathic systemic tumor lysis syndrome was described by Crittenden and Ackerman in 1977 [8]. The patient presented with a one-month history of anorexia, abdominal fullness (secondary to ascites), gingival bleeding, bilateral leg swelling, and weight loss; initial laboratory studies showed a hyperkalemic metabolic acidosis and a liver scan showed multiple filling defects consistent with metastatic disease. He subsequently developed hyperuricemic acute renal failure and died; his autopsy showed metastatic poorly differentiated, anaplastic adenocarcinoma in the liver, abdominal lymph nodes, and vertebral column without identification of a primary tumor [8].

Solid tumor-associated spontaneous (idiopathic) systemic tumor lysis syndrome has subsequently been observed in patients with various cancers. These include adenocarcinoma of unknown primary, breast carcinoma, colorectal carcinoma, endometrial carcinoma, gastric carcinoma, germ cell tumors, hepatocellular carcinoma, lung cancer (non-small cell and small cell), melanoma, prostate carcinoma, renal cell carcinoma, and rhabdomyosarcoma (embryonal and parietal) [2,7,8,10,11,15-23].

Therapy-Associated Systemic Tumor Lysis Syndrome

Therapy-associated systemic tumor lysis syndrome occurs in patients with a known diagnosis of cancer. There is lysis of the tumor cells following the initiation of malignancy-directed therapy. The treatments associated with the subsequent development of systemic tumor lysis syndrome include chemotherapy, corticosteroids, embolization, immunotherapy, interferon, monoclonal antibodies, radiotherapy, surgery, and tamoxifen [1,2,9-11,15,17,20,21,23-26].

Hematologic malignancies: Therapy-associated systemic tumor lysis syndrome was initially observed in patients with hematologic malignancies. Indeed, the reported incidence has ranged from 42% to 4%. However, an increased awareness by oncologists of therapy-associated systemic tumor lysis syndrome and their use of preventative measures resulted in a significant decrease in the incidence of this subtype of systemic tumor lysis [1,2,9,10].

Risk factors for the development of therapy-associated systemic tumor lysis syndrome in patients with hematologic cancers include malignancies with a greater cancer burden (manifested by increased tumor mass or involvement of other organs and bone marrow or both), malignancies that are rapidly proliferating, and malignancies that are highly sensitive to chemotherapy. Hence, patients with either high-grade, aggressive, and bulky lymphomas (such as Burkitt lymphoma and acute lymphoblastic lymphoma) or acute leukemias (such as acute lymphocytic leukemia or acute myelogenous leukemia with white blood cell counts greater than 100,000/µL) are more susceptible to therapy-associated systemic tumor lysis syndrome. In addition, dehydration and diminished kidney function (which may be more common in older individuals) have also been associated with an increased risk of systemic tumor lysis syndrome developing after malignancy-directed therapy has been initiated [1,2,9,10].

Solid tumors: Similar to solid tumor-associated spontaneous (idiopathic) systemic tumor lysis syndrome, therapy-associated systemic tumor lysis syndrome in patients with solid tumors is uncommon. However, there appears to be an increasing number of cancer patients with visceral malignancies in whom therapy-associated systemic tumor lysis syndrome is being reported. Solid tumors in which therapy-associated systemic tumor lysis syndrome has been observed include adenocarcinoma of unknown primary, breast cancer (infiltrating ductal carcinoma), colorectal carcinoma, gastric adenocarcinoma, hepatoblastoma, hepatocellular carcinoma, lung cancer (non-small cell carcinoma and small cell carcinoma), medulloblastoma, melanoma, neuroblastoma, pancreatic cancer (neuroendocrine carcinoma), ovarian carcinoma, prostate cancer (adenocarcinoma and small cell carcinoma), sarcoma, uterine cancer (serous carcinoma), and vulvar carcinoma [10,11,15,17,20,21,23-26].

Cutaneous tumor lysis syndrome

We suggest that cutaneous tumor lysis syndrome refers to the lysis of neoplastic cells located within the dermis from either hematologic malignancies (such as lymphomas) or solid tumors (such as cutaneous metastases). Similar to systemic tumor lysis syndrome, cutaneous tumor lysis syndrome can be spontaneous (idiopathic) or therapy-associated. Clinically, cutaneous tumor lysis syndrome presents as ulcers (which have also been described as tumor necrosis) within the tumor mass. A critical criteria for cutaneous tumor lysis syndrome is the presence of rapid tumor cell death [3-5,7,27-50].

Spontaneous (Idiopathic) Cutaneous Tumor Lysis Syndrome

There are multiple cases in the literature suggesting that spontaneous (idiopathic) cutaneous tumor lysis syndrome may occur in hematologic malignancies (such as B-cell lymphomas and T-cell lymphomas). The key feature is necrosis and ulceration of the skin tumors in the absence of therapy. In addition, albeit rare to date, cases suggestive of this diagnosis have been observed in patients with untreated solid tumors (such as melanoma, breast cancer, and Kaposi sarcoma) [7,27-39].

It is possible that the appearance of spontaneous (idiopathic) cutaneous tumor lysis syndrome may occur following the rapid peripheral proliferation and growth of the cutaneous lymphoma or visceral tumor metastases associated with the concurrent-pronounced and expedient-death of the cancer cells. Lysis of the tumor cells may occur as a result of inadequate vascularization to the central area of the neoplasm. There is subsequent necrosis and ulcer formation as the clinical presentation of spontaneous (idiopathic) cutaneous tumor lysis syndrome. However, it remains unclear if some of these cases were accompanied by rapid tumor death, a key feature of cutaneous tumor lysis syndrome [7,27-39].

Hematologic malignancies: Hematologic malignancies associated with features suggestive of spontaneous (idiopathic) cutaneous tumor lysis syndrome include both T-cell and B-cell neoplasms. The T-cell neoplasms include mycosis fungoides, extranodal natural killer/T-cell lymphoma, nasal type, and cutaneous peripheral T-cell lymphoma of cytotoxic phenotype (Table 2) [27-30]. The B-cell neoplasms of patients possibly presenting with characteristics of spontaneous (idiopathic) cutaneous tumor lysis syndrome include cutaneous B-cell lymphoma, intravascular B-cell lymphoma, primary cutaneous Epstein-Barr virus-positive diffuse large B-cell lymphoma, not otherwise specified, and cutaneous lymphomatoid granulomatosis (Table 3) [31-34].

**Table 2:** Examples of T-cell neoplasm-associated possible spontaneous (idiopathic) cutaneous tumor lysis syndrome. CPTCL-CP, cutaneous peripheral T-cell lymphoma of cytotoxic phenotype; ENK/TCL, extranodal natural killer/T-cell lymphoma; Ref, reference; yo, year old

Patients	Tumor	Comments	Ref
14 of 50 patients	Mycosis fungoides	In a study of 50 patients with primary and recurrent mycosis fungoides, 14 had either erythroderma and/or ulcerated lesions, on initial presentation; some of the patients with ulcerated lesions might be compatible with spontaneous (idiopathic) cutaneous tumor lysis syndrome	[27]
65 yo man	Mycosis fungoides	Necrotizing mycosis fungoides in a therapy-naive man may also represent spontaneous (idiopathic) cutaneous lysis syndrome	[28]
51 yo man	ENK/TCL, nasal type	ENK/TCL, nasal type presented in a previously healthy man with possible spontaneous (idiopathic) cutaneous tumor lysis syndrome as painless, ulcerated cutaneous lesions over his trunk and limbs	[29]
55 yo man	CPTCL-CP	CPTCL-CP presented with possible spontaneous (idiopathic) cutaneous tumor lysis syndrome-type characteristics as an ulcerated 5-cm cutaneous plaque on a man’s left ankle. During the subsequent month, nine additional, similar appearing, ulcerated plaques developed on his trunk and bilateral extremities	[30]

**Table 3:** Examples of B-cell neoplasm-associated possible spontaneous (idiopathic) cutaneous tumor lysis syndrome. EBV, Epstein Barr virus; LG, lymphomatoid granulomatosis; LPD, lymphoproliferative disorder; Ref, reference; yo, year old

Patients	Tumor	Comments	Ref
56 yo man	Cutaneous B-cell lymphoma	A man presented with a rapidly growing tumor with satellite lesions of two-month duration on his upper back; the possible spontaneous (idiopathic) cutaneous tumor lysis manifested as skin necrosis with hemorrhage, suppuration, and ulceration of the 8 × 8-cm tumor mass	[31]
76 yo woman	Intravascular B-cell lymphoma	A woman’s lymphoma presented as possible spontaneous (idiopathic) cutaneous tumor lysis syndrome with two-month history of anorexia, fever, malaise, weight loss, and necrotic and crusted papules on her thighs	[32]
35 yo man	EBV- associated B-cell LPD	An immunocompetent man’s primary cutaneous, EBV-positive diffuse large B-cell lymphoma, not otherwise specified, presented with possible spontaneous (idiopathic) cutaneous tumor lysis; during the prior six months prior to medical evaluation, his lymphoma appeared as multiple, painful, and ulcerated nodules on his trunk and arms	[33]
Two of 20 patients	Cutaneous LG	At least two of 20 patients with cutaneous LG, an angiocentric and angiodestructive EBV-associated B-cell lymphoproliferative disorder, had lesions that were suggestive of spontaneous (idiopathic) cutaneous tumor lysis; their dermal and subcutaneous nodules have necrosis and central ulceration	[34]

Solid tumors: In addition to patients with Kaposi sarcoma, characteristics possibly compatible with solid tumor-associated spontaneous (idiopathic) cutaneous tumor lysis syndrome have also been reported in patients with other solid tumors (Table 4) [7,35-39]. For example, a 79-year-old man with metastatic melanoma in whom the phenomenon was referred to as giant centrifugal and necrotizing cutaneous metastases of melanoma [35]. Another patient was a woman with metastatic breast cancer who concurrently presented with not only spontaneous (idiopathic) cutaneous tumor lysis syndrome but also spontaneous (idiopathic) systemic tumor lysis syndrome [39].

**Table 4:** Examples of possible solid tumor-associated spontaneous (idiopathic) cutaneous tumor lysis syndrome. Ref, reference; yo, year old

Patients	Tumor	Comments	Ref
Four men	Kaposi sarcoma	Kaposi sarcoma may also present with ulcerated or necrotic nodules that can suggest spontaneous (idiopathic) cutaneous tumor lysis syndrome. The ulcerated and necrotic tumors can appear in patients with either classic or human immunodeficiency virus-associated Kaposi sarcoma and present as unusual penile ulcers or mimic diabetic foot ulcers	[36-39]
79 yo man	Melanoma	A man originally presented with an ulcerated nodular malignant melanoma (Breslow thickness was 4.5 mm and the mitotic rate was eight mitoses/mm^2^) on his right scapular region that was excised. Subsequently, his melanoma-associated possible spontaneous (idiopathic) cutaneous tumor lysis syndrome appeared as a rapidly progressing large, odorous, bleeding metastatic tumor nodule with central necrosis, which measured 32 × 40 × 10 cm, on his upper back	[35]
36 yo woman	Breast cancer	A woman presented with a 6-7-cm fungating erythematous left breast mass with irregular margins and a necrotic surface with foul-smelling purulent discharge that had eroded through her skin. In addition to invasive ductal breast carcinoma-associated possible spontaneous (idiopathic) cutaneous tumor lysis syndrome, the rapid tumor cell death also resulted in solid tumor-associated spontaneous (idiopathic) systemic tumor lysis syndrome with associated hyperuricemia, hyperkalemia, hyperphosphatemia, and elevation of both creatinine and lactate dehydrogenase	[7]

The investigators speculated that the woman’s problems resulted from the rapid cancer cell death and necrosis of her large local breast tumor. Her spontaneous (idiopathic) cutaneous tumor lysis syndrome occurred once the tumor had outgrown its blood supply. The ischemic insult to the cancer resulted in not only ulceration of the massive cutaneous metastasis but also release of the intracellular contents of the tumor cells, with the subsequent laboratory and clinical features of spontaneous (idiopathic) systemic tumor lysis syndrome [7].

Therapy-Associated Cutaneous Tumor Lysis Syndrome

Therapy-associated cutaneous tumor lysis syndrome may be more prevalent than the number of reported individuals would suggest. Features suggestive of this syndrome have been observed in oncology patients with hematologic malignancies such as lymphomas. Cancer patients whose solid tumors have characteristics compatible with therapy-associated cutaneous tumor lysis syndrome include individuals with Kaposi sarcoma, women with metastatic breast cancer, and our patient with metastatic salivary duct carcinoma [3-5,27,40-50].

Treatments associated with possible cutaneous tumor lysis syndrome in patients with T-cell lymphoma include either total skin electron irradiation or antineoplastic therapies. The latter include either bexarotene, vorinostat and fenofibrate, or retinoids (etretinate or acitretin) and subcutaneous interferon alpha-2b. In addition, methotrexate has been associated with therapy-associated cutaneous tumor lysis syndrome in patients with either T-cell lymphoma or B-cell lymphoma [27,40-43].

Possible therapy-associated cutaneous tumor lysis syndrome has been observed in Kaposi sarcoma patients who have been treated with either radiotherapy, vinblastine, or vincristine [44-46]. Our patient with metastatic salivary duct carcinoma developed rapid death of his tumor cells, manifesting clinically as necrosis and ulcers at the location of his skin metastases, within days after his first infusion of bevacizumab and temsirolimus. Because he had phosphatase and tensin homolog (PTEN) loss [resulting in activation of the phosphatidylinositol 3-kinase (PI3K) pathway, which is targeted by mechanistic target of rapamycin (mTOR) inhibitors such as temsirolimus], we postulate that temsirolimus alone or in synergy with bevacizumab resulted in his dramatic response to treatment [3-5].

Bevacizumab, as a component of combination antineoplastic treatment, was associated with therapy-associated cutaneous tumor lysis syndrome in several patients with metastatic breast carcinoma. Women were often also being treated with paclitaxel or docetaxel. However, the other agents they were concurrently receiving included vinorelbine, or capecitabine, or cyclophosphamide and methotrexate [47-49].

Hematologic malignancies: Features of therapy-associated cutaneous tumor lysis syndrome have been observed in patients with either T-cell or B-cell hematologic malignancies (Tables 5, 6) [27,40-43]. Investigators postulated that the extensive necrosis of mycosis fungoides tumors in a 50-year-old man were caused by treatment-induced increased rate of apoptosis in the lymphoma cells and the subsequent release of lymphoma antigens. The augmented release of tumor antigens, in conjunction with changes in the local cytokine milieu, resulted in the central necrosis of his fully developed edematous elevated lymphoma [42].

**Table 5:** Examples of T-cell hematologic malignancy-related possible therapy-associated cutaneous tumor lysis syndrome. CD8, cluster of differentiation 8; PCAEC, primary cutaneous aggressive epidermotropic cytotoxic; Ref, reference; yo, year old; +, positive

Patients	Tumor	Comments	Ref
Three of 50 patients	Mycosis fungoides	In a study of 50 patients with primary and recurrent mycosis fungoides, only eight of the 14 patients who had either erythroderma and/or ulcerated lesions on initial presentation developed “severe moist desquamation” during total skin electron irradiation; three of the 50 patients were hospitalized due to possible therapy-associated cutaneous lysis syndrome appearing as ulceration and skin infection	[27]
50 yo man	Mycosis fungoides	A man with only cutaneous tumor stage mycosis fungoides developed painful extensive necrosis of the lymphoma lesions, compatible with possible therapy-associated cutaneous tumor lysis syndrome, 18 weeks after beginning treatment with bexarotene, vorinostat, and high-dose fenofibrate	[42]
45 yo man	CD8+ mycosis fungoides	A man with hypopigmented CD8+ mycosis fungoides and possible therapy-associated cutaneous tumor lysis syndrome who developed central necrosis of existing lesions and new lesions that would ulcerate after initiating treatment with systemic retinoids (either etretinate or acitretin) and subcutaneous interferon alpha-2b	[41]
68 yo man	CD8+ PCAEC T-cell lymphoma	A man with CD8+ PCAEC T-cell lymphoma developed therapy-associated ulceration and necrosis of the new skin lesions compatible with possible therapy-associated cutaneous lysis syndrome after methotrexate was started	[40]

**Table 6:** Example of B-cell hematologic malignancy-related possible therapy-associated cutaneous tumor lysis syndrome. DLCL, diffuse large cell lymphoma; EBV, Epstein-Barr virus; Mtx, methotrexate; PC, primary cutaneous; R-CHOP, rituximab, cyclophosphamide, vincristine, doxorubicin, and prednisone; Ref, reference; yo, year old

Patients	Tumor	Comments	Ref
67 yo woman	Mtx-induced EBV-negative PC T-cell rich B-cell DLCL	A woman, receiving weekly oral Mtx for rheumatoid arthritis, developed painless, disseminated, erythematous patches and nodules on her trunk, face, and limbs. The possible Mtx-associated cutaneous tumor lysis syndrome presented as necrotic centered skin lesions that increased with time and progressed to ulcer formation. All lesions eventually resolved without recurrence after Mtx was discontinued and she completed eight cycles of her new therapy with R-CHOP	[43]

Solid tumors: Signs and symptoms suggestive of therapy-associated cutaneous tumor lysis syndrome have been observed in Kaposi sarcoma patients; they occurred following treatment with either radiotherapy or intralesional agents such as vinblastine and vincristine (Table 7) [44-46]. Therapy-associated cutaneous tumor lysis syndrome has also been observed in breast cancer patients with extensive skin metastases after initiation of combination therapy which included bevacizumab; the phenomenon was described as either skin necrosis or tumor necrosis or large ulcer expansion, and it occurred shortly after the first cycle of treatment with bevacizumab-based therapy (Table 8) [47-49]. In addition, we observed therapy-associated cutaneous tumor lysis syndrome in a man whose metastatic salivary duct carcinoma demonstrated rapid cell death and presented as necrosis and ulcers within days after receiving his initial cycle of dual-agent (bevacizumab and temsirolimus) antineoplastic therapy [3-5].

**Table 7:** Examples of Kaposi sarcoma-related possible therapy-associated cutaneous tumor lysis syndrome. Ref, reference; yo, year old

Patients	Comments	Ref
10 of 131 evaluable patients	A retrospective study of radiotherapy in 149 individuals with cutaneous AIDS-related epidemic Kaposi sarcoma had 131 evaluable patients; 10 (8%) of the patients developed possible therapy-associated cutaneous tumor lysis syndrome with skin ulcerations and severe epidermitis	[44]
One of six men	A study of intralesional vinblastine for the treatment of plaques and nodules of Kaposi sarcoma of at least 2.5 cm was performed. One of the six men developed possible therapy-associated cutaneous tumor lysis syndrome presenting as central necrosis that occurred at the vinblastine treatment site and progressed to a deep ulceration	[45]
Three of 151 patients	Intralesional vincristine was studied in the treatment of 151 patients with stage IB classical Kaposi sarcoma nodular lesions. Three (2%) patients developed manifestations compatible with therapy-associated cutaneous tumor lysis syndrome; they experienced grade 3 reactions demonstrating severe pain, erythema, bullae, and ulceration	[46]

**Table 8:** Examples of breast cancer-related possible therapy-associated cutaneous tumor lysis syndrome. Ref, reference; yo, year old

Patients	Comments	Ref
Nine of 12 women	A retrospective study described 12 women with advanced infiltrating ductal breast carcinoma who had extensive cutaneous metastases and were treated with antineoplastic therapy that included bevacizumab. In nine women, patches of enlarging skin erosions developed at the cutaneous metastasis sites. In three women, the erosions remained superficial, while there was progressive peripheral growth of the erosions. However, in six women, large areas of complete skin layer loss resulted in the erosions progressing to deep ulcers and necrosis, features suggestive of cutaneous tumor lysis syndrome, with exposure of the underlying fat and muscle. The investigators concluded that bevacizumab-associated necrosis and ulceration in women with breast cancer treated with this agent was usually related to extensive tumor involvement of the skin	[47]
27 yo woman	A woman presented with metastatic invasive ductal breast carcinoma (scirrhous carcinoma) began her seventh-line therapy with bevacizumab and paclitaxel. Her nodular and partially erosive left breast tumor measured 15 × 12 × 9 cm. Within three days, there was therapy-associated cutaneous tumor lysis syndrome manifested by necrosis of all skin layers and the development of deep ulcers that exposed the pectoralis major muscle. A second treatment cycle was started; however, she died from carcinomatous lymphangitis-associated respiratory disorder. These observations suggest local (cutaneous) tumor death without systemic regression	[48]
48 yo woman	A woman with metastatic invasive ductal breast carcinoma (scirrhous carcinoma) began her third-line therapy with bevacizumab and paclitaxel. Prior to this treatment, the left chest wall had a 5-cm ulcerative lesion. After two treatment cycles, the entire cutaneous breast cancer lesion detached; the residual ulcer was 20 × 15 cm, and not only the chest wall but also the underlying muscles (pectoralis major, pectoralis minor, and anterior serratus muscle) had disappeared and part of her rib was exposed	[49]

Bevacizumab is a humanized monoclonal antibody that selectively binds to vascular endothelial growth factor-A. Vascular endothelial growth factor is a glycoprotein that stimulates angiogenesis; this can occur not only by the proliferation of blood vessels from preexisting normal vasculature but also by the enhancement of abnormal tumor vasculature. Hence, bevacizumab has previously been used as a treatment component for patients with glioblastoma multiforme and metastatic carcinoma [such as breast (no longer approved), colon, non-small-cell lung, and renal cell cancer] [50].

Bevacizumab-associated side effects include arterial thromboembolism, hemorrhage, hypersensitivity reactions, hypertension, and gastrointestinal perforation. In addition, because vascular endothelial growth factor is involved in proper wound healing, treatment with bevacizumab has been observed to cause wound healing complications. Therefore, bevacizumab therapy is withheld for a period of time before and after surgery [50].

Our patient was a 70-year-old man with metastatic salivary duct carcinoma (a disease pathologically reminiscent of ductal breast cancer) developed what appeared to be therapy-associated cutaneous tumor lysis syndrome after receiving his first cycle of bevacizumab and temsirolimus. Eleven months earlier, his salivary duct carcinoma presented as a left preauricular parotid gland mass. His disease progressed to involve his left axilla and chest wall [3-5].

**Figure 1:**
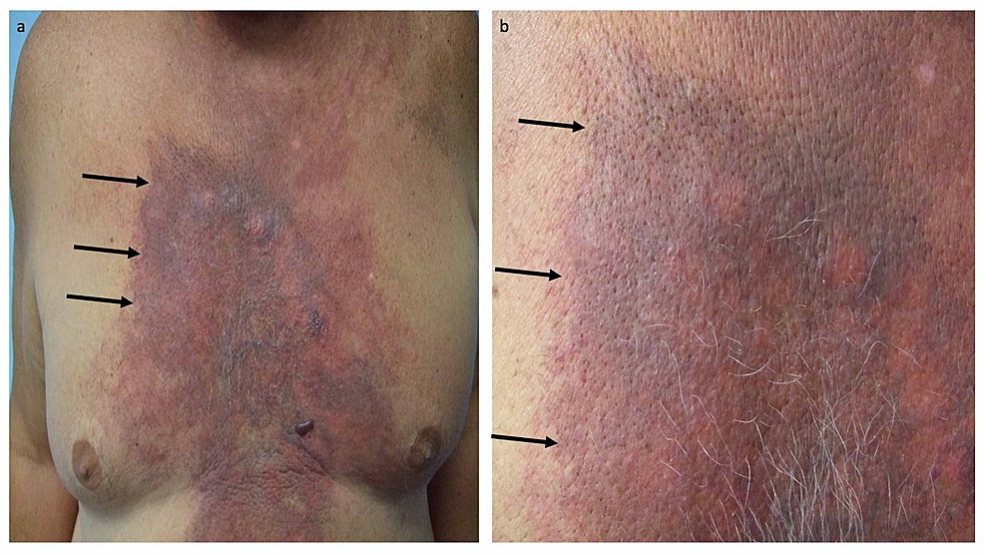
Cutaneous metastatic salivary duct carcinoma-associated carcinoma hemorrhagiectoides presenting as the shield sign. A 70-year-old man with metastatic salivary duct carcinoma appearing as shield sign-associated carcinoma hemorrhagiectoides presented 11 months earlier with a left preauricular parotid gland mass. Additional lesions developed in his left axilla and chest wall within two months. Both the parotid gland mass and left axillary lymph node showed salivary duct carcinoma. He was treated with five cycles of docetaxel followed by four weeks of chemoradiation (45 Gray in 20 fractions and weekly cetuximab). Two months after completing chemoradiation, he presented with progressive neoplastic disease not only involving lymph nodes of the mediastinum and both axillae but also cutaneous metastases. Distant (a) and closer (b) views of his cutaneous metastatic salivary duct carcinoma appeared as a hemorrhagic and erythematous confluent large violaceous and purpuric plaque (black arrows) that extended from his neck to his abdomen and across his chest, resembling a medieval knight’s shield. The images have not previously been published; however, the details of the patient’s skin lesions and clinical course have previously been described [3-5].

He initially showed a good response to treatment with docetaxel which was followed by chemoradiation. However, two months later, he presented with progressive neoplastic disease not only involving lymph nodes of the mediastinum and both axillae but also cutaneous metastases (carcinoma hemorrhagiectoides that appeared as a hemorrhagic and erythematous confluent large violaceous and purpuric plaque) that extended from his neck to his abdomen and across his chest. The skin metastases resembled a medieval knight’s shield (Figure 1) [3-5].

**Figure 2:**
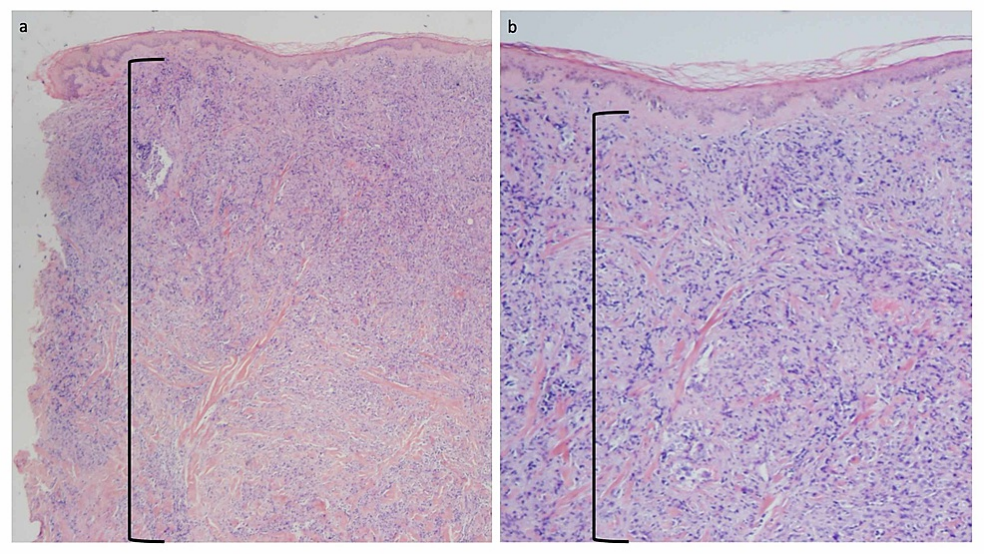
Microscopic examination of cutaneous metastatic salivary duct carcinoma. Low (a) and higher (b) magnification views of the punch biopsy in the hemorrhagic area of the chest are shown. The depth of the biopsy was 10 mm and extended into the deep reticular dermis; the deep margin of the skin biopsy did not reach the subcutaneous fat. Microscopic examination of the tissue specimen shows a narrow zone of normal-appearing papillary dermis beneath the epidermis. Below this narrow zone, there is a confluent and dense infiltrate of metastatic carcinoma cells that are not only present in the dermal vessels but also extend to the base of the specimen in the deep reticular dermis (demonstrated by the area demarcated by the black open bracket (hematoxylin and eosin: a, ×20; b, ×40). The images have not previously been published; however, the details of the pathology of the patient’s skin lesions have previously been described [3-5].

A skin biopsy of the chest cutaneous metastasis was performed to a depth of 10 mm. It demonstrated the metastatic salivary duct carcinoma tumor cells had replaced the entire dermis. There was an extensive, confluent and dense, infiltrate of metastatic tumor cells that extended from the upper reticular dermis to the base of the specimen in the deep reticular dermis (Figure 2) [3-5].

**Figure 3:**
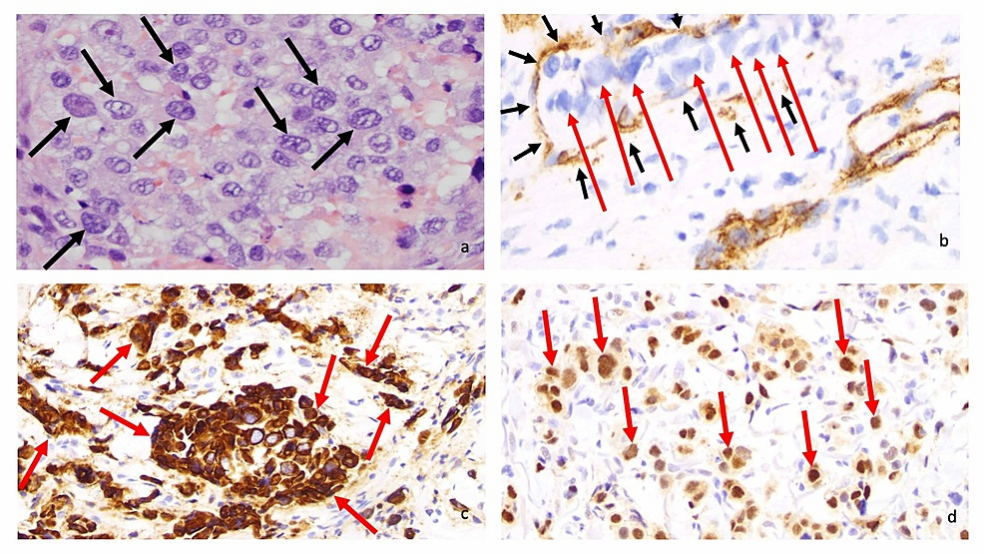
Microscopic examination of cutaneous metastatic salivary duct carcinoma. Microscopic examination at high magnification of the neoplastic salivary duct carcinoma cells shows large, irregular nuclei (black arrows) with visible nucleoli (a). Immunohistochemistry (brown staining) with anti-cluster of differentiation 31 (anti-CD31) highlights vascular endothelial cells (black arrows) containing neoplastic cells (red arrows), thereby demonstrating vascular invasion by the metastatic carcinoma (b). Anti-cytokeratin 7 (anti-CK7) and androgen receptor (both brown staining) label tumor cells (red arrows) (c and d, respectively) (a, hematoxylin and eosin, ×400; b, anti-CD31, diaminobenzidine immunoperoxidase and light hematoxylin, x 400; c, anti-CK7, diaminobenzidine immunoperoxidase and light hematoxylin, ×400; anti-androgen receptor, diaminobenzidine immunoperoxidase and light hematoxylin, ×400). The images have not previously been published; however, the details of the pathology of the patient’s skin lesions have previously been described [3-5].

The nuclei of the neoplastic salivary duct carcinoma cells were large and irregular; the nucleoli were also visible. The metastatic carcinoma tumor cells demonstrated vascular invasion; they were observed within the dermal vessels whose vascular endothelial cells were stained by anti-cluster of differentiation 31 (anti-CD31). The tumor cells expressed both cytokeratin 7 (CK7) and androgen receptor, confirming the diagnosis of salivary duct carcinoma (Figure 3) [3-5].

**Figure 4:**
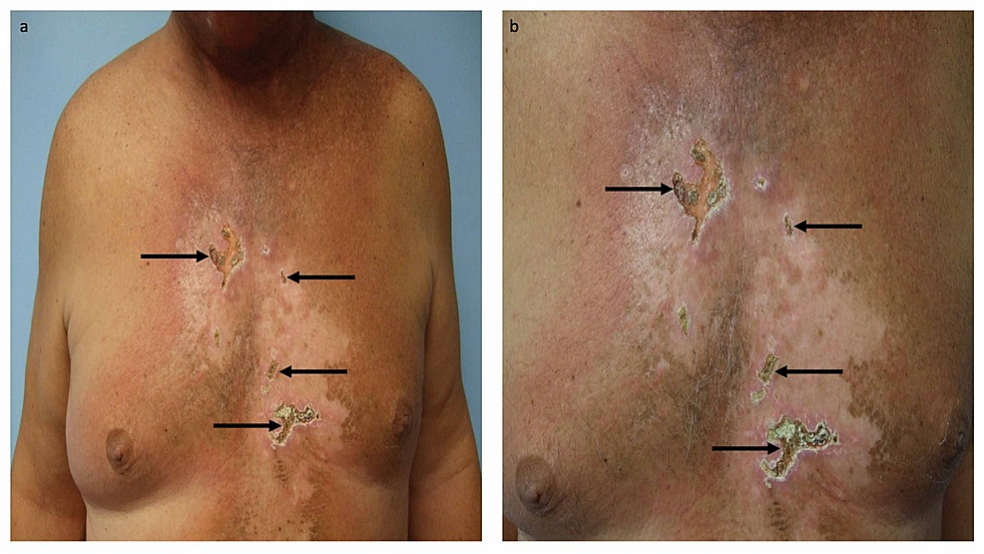
Cutaneous tumor lysis syndrome following treatment with temsirolimus and bevacizumab. After the patient had progressed on chemoradiation, he was treated with temsirolimus (at a dose of 25 mg intravenously on days one, eight, and 15) and bevacizumab (at a dose of 15 mg/kg on day one) on 21 day therapy cycles. Distant (a) and closer (b) views show therapy-associated cutaneous tumor lysis syndrome presenting as several areas of full thickness ulcers (black arrows) on his chest that he experienced after his first cycle of treatment; the other areas of his chest and abdomen also demonstrated the dramatic resolution of his cutaneous metastases. The images have not previously been published; however, the details of the patient’s skin lesions and clinical course have previously been described [3-5].

He was treated with bevacizumab (at a dose of 15 mg/kg on day one) and temsirolimus (at a dose of 25 mg intravenously on days one, eight, and 15) on 21 day therapy cycles. Within a few days after receiving the first dose of bevacizumab and temsirolimus, therapy-associated cutaneous tumor lysis syndrome occurred. Several areas of full thickness ulcers developed on his chest. In addition, he also experienced a dramatic resolution of his cutaneous metastases on the other areas of his chest and abdomen (Figure 4) [3-5].

We originally observed his cutaneous manifestations a few years prior to the observation of bevacizumab-based therapy-associated similar cutaneous signs in women with metastatic breast cancer, a disease remarkably similar to salivary duct cancer [47-49]. Our initial (and current) hypothesis was that the man’s salivary duct carcinoma had an exceptional therapeutic response to the antineoplastic activity of either the bevacizumab or the temsirolimus or both agents; in addition to his skin metastases showing a remarkable response to the therapy, his subcarinal lymph nodes also demonstrated a dramatic 62% decrease in volume [3-5].

Individuals with salivary duct carcinoma can have phosphatidylinositol-4,5-bisphosphate 3-kinase catalytic subunit alpha (PIK3CA) mutations, which would sensitize the tumor to the mTOR inhibitor temsirolimus. In addition, patients who have tumors with PTEN loss also activate the PI3K pathway which is targeted by mTOR inhibitors. Our patient had no PIK3CA mutation; however, he had PTEN loss. To our knowledge, bevacizumab, as a single agent, does not have activity in salivary duct carcinoma and therefore would not be responsible for the rapid tumor regression that occurred and is an essential feature of cutaneous tumor lysis syndrome. Therefore, the rapid tumor regression in our patient may be due to the temsirolimus instead of the bevacizumab or possibly due to synergy from the combination.

Because, as shown on the skin biopsy of our patient, his chest cutaneous metastases showed a complete replacement of the normal dermis by salivary duct carcinoma tumor cells, an exuberant therapeutic response that lysed all of the cutaneous tumor cells that had previously filled the dermis and would result in a complete absence of the dermal tissue between the subcutaneous fat and the residual overlying epidermis. Subsequently, the epidermis would promptly become devitalized and shed. This would expose an ulcer created by the absent underlying dermis.

A second, not mutually exclusive, hypothesis for the development of cutaneous tumor lysis syndrome not only in the patients with metastatic breast cancer but also in our patient with metastatic salivary duct carcinoma is related to the anti-angiogenesis activity of bevacizumab. Indeed, it may be more than mere coincidence that all of these oncology patients with solid tumors developed therapy-associated cutaneous tumor lysis syndrome after treatment with a bevacizumab-based antineoplastic regime was initiated. It is reasonable to postulate that bevacizumab’s ability to block the activity of dermal vessel vascular endothelial growth factor may have resulted in sufficient vascular damage to result in anoxia and subsequent lysis of the metastatic tumor cells in the dermis and the prompt development of skin ulcers where the cutaneous metastases had previously been located.

## Conclusions

Tumor lysis syndrome has been defined by laboratory and clinical criteria; it occurs more frequently in oncology patients with hematologic malignancies than those with solid tumors. In these cancer patients, tumor lysis syndrome is typically precipitated by effective neoplasm-directed therapy; however, the syndrome can present spontaneously, for reasons deemed idiopathic, prior to the individual receiving malignancy-directed treatment. We previously described a man with metastatic salivary duct carcinoma whose cutaneous metastases presented as a dermal infiltration of the tumor cells which became necrotic and developed into ulcers after he received his first dose of antineoplastic therapy. The dramatic response to his treatment and finding of multiple analogous reports in the literature have prompted us to suggest a modification to the classification of tumor lysis (or necrosis) syndrome. Our observations and those in the literature suggest that, in addition to the well-established traditional tumor lysis syndrome, which we herein designate “systemic tumor lysis syndrome,” there exists a “cutaneous tumor lysis syndrome,” which manifests by substantial necrosis and ulceration of the skin malignancy in the presence of rapid tumor cell death. We further suggest that cutaneous tumor lysis syndrome can be subclassified into therapy-associated and spontaneous (idiopathic) subtypes. Although manifestations of cutaneous tumor lysis syndrome such as extensive ulceration and necrosis have been mainly observed after treatment (therapy-associated cutaneous tumor lysis syndrome), there are some reports compatible with a spontaneous (idiopathic) cutaneous tumor lysis syndrome, analogous to the spontaneous (idiopathic) systemic tumor lysis syndrome. With improvement in therapy, especially for patients with extensive metastatic disease, therapy-associated cutaneous tumor lysis syndrome may be reported more frequently, and supportive measures to heal the skin may be necessary.
